# Characterisation and Modelling of Ultrashort Laser-Driven Electromagnetic Pulses

**DOI:** 10.1038/s41598-020-59882-8

**Published:** 2020-02-20

**Authors:** Kwinten Nelissen, Máté Liszi, Massimo De Marco, Valeria Ospina, István Drotár, Giancarlo Gatti, Christos Kamperidis, Luca Volpe

**Affiliations:** 1 ELI-ALPS, ELI-HU Non-Profit Ltd., Dugonics tér 13, Szeged, 6720 Hungary; 2 Centro de Laseres Pulsados (CLPU), Calle del Adaja 8, Villamayor, 37185 Salamanca, Spain; 3 Laser-Plasma Chair at the University of Salamanca, Salamanca, Spain; 4 Radiofrequency Test Laboratory (RFTL) - Széchenyi István University, Egyetem tr 1, Gyor, 9026 Hungary

**Keywords:** Physics, Laser-produced plasmas

## Abstract

Recent advances on laser technology have enabled the generation of ultrashort (fs) high power (PW) laser systems. For such large scale laser facilities there is an imperative demand for high repetition rate operation in symbiosis with beamlines or end-stations. In such extreme conditions the generation of electromagnetic pulses (EMP) during high intense laser target interaction experiments can tip the scale for the good outcome of the campaign. The EMP effects are several including interference with diagnostic devices and actuators as well as damage of electrical components. The EMP issue is quite known in the picosecond (ps) pulse laser experiments but no systematic study on EMP issues at multi-Joule fs-class lasers has been conducted thus far. In this paper we report the first experimental campaign for EMP-measurements performed at the 200 TW laser system (VEGA 2) at CLPU laser center. EMP pulse energy has been measured as a function of the laser intensity and energy together with other relevant quantities such as (i) the charge of the laser-driven protons and their maximum energy, as well as (ii) the X-ray K_*α*_ emission coming from electron interaction inside the target. Analysis of experimental results demonstrate (and confirm) a direct correlation between the measured EMP pulse energy and the laser parameters such as laser intensity and laser energy in the ultrashort pulse duration regime. Numerical FEM (Finite Element Method) simulations of the EMP generated by the target holder system have been performed and the simulations results are shown to be in good agreement with the experimental ones.

## Introduction

## Results

High intensity laser pulses are shown to be capable of generating intense broadband, i.e. from few hundreds of MHz up to THz^1^, electromagnetic pulses (EMP’s) through different regimes of interactions^2–6^. When high intense laser target interaction takes place^7–9^, a significant portion of electrons escape the target and a fraction of those electrons are accelerated towards relativistic energies generating an ultrashort burst of electromagnetic pulse (EMP) as given by the Lienard Wiechert potentials^10–15^. Moreover, the remaining positive charge^16^ on the target drives a re-circulating current in the vicinity of the laser spot that gives rise to a THz radiation. Finally, neutralizing currents flow inside the target and target holder which last for a time scale of several orders of magnitudes longer than the laser pulse duration. These currents lead to the emission of Radio Frequency radiation in the frequency range from MHz towards multiple of GHz (see ref. ^4^).

The EMP propagating inside and outside the target vacuum chamber^17^, can interfere with power cables and diagnostics leading to perturbation or disruption of signal communications as well as loss of experimental data. With the upcoming of new high-repetition rate, high-power, short pulse user facilities, like the Extreme Light Infrastructure ALPS^18^, it becomes imperative to find strategies for mitigation and control of EMP and related detrimental effect. Mitigation options include: shielding of electronic devices with Faraday cages; using shielded cables; disabling and electrically decoupling devices during the laser shot; placing sensitive equipment far away from the interaction area; development of a dedicated grounding for susceptible devices, shielding the target^19^ and ensuring a good electrical isolation of the target. On the other hand the understanding of physical phenomena behind the generation of EMP, could lead to new generation laser driven radio-frequency (RF) sources, which is of strong interest for space industries (satellite instrumentation protection) and novel fusion energy schemes^20,21^.

The EMP features are strongly related to the target and chamber geometry. In fact the target holder system behaves as an emitting antenna and the vacuum chamber acts in principle as a resonance cavity^22,23^. Measurements conducted at different laboratories (See Fig. 4 in ref. ^24^ have demonstrated extremely high electrical field strengths of the EMP’s under comparable laser pulse intensities^25–27^: up to 100’s of kV/m peak to peak while return currents through the target holder can achieve amplitudes of 10’s of kA. Moreover, A. Poye *et al*.^4^ identified three different interaction regimes which rules the discharge mechanism, i.e. the full ejection regime, the thermal regime, and the quasi-steady state regime. In the full ejection regime, which is applicable for ultrashort laser pulses, the discharge time is orders of magnitude larger than the duration of the laser pulse.

Following ref. ^4^, the EMP generated by the fs laser-target interaction at VEGA 2, can be described by the "Full ejection regime” in which most of the laser-accelerated electrons can escape the potential barrier and their average temperature ⟨*T*_*e*_⟩ is directly related to the Laser Intensity $\langle {T}_{e}\rangle  \sim {I}_{L}^{1/2,1/3}$. In this regime one can estimate that the total electrical charge on target is *Q*_*h*_ ~ *eN*_*e*_, assuming: (i) the conservation of energy *E*_*L*_*η*_*L*,*e*_ = *N*_*e*_⟨*T*_*e*_⟩ (where *E*_*L*_ is the laser energy, *N*_*e*_ is the electron number and *η*_*L*,*e*_ is the conversion efficiency between laser and hot electron population) and (ii) *E*_*EMP*_ ∝ *Q*_*h*_. Consequently, there is a direct relation between *E*_*EMP*_ and laser Intensity or between *E*_*EMP*_ and laser energy. In addition *E*_*EMP*_ is related to the interaction volume given by Δ*t*Δ*A* (A $\sim {r}_{L}^{2}$, where *r*_*L*_ is the radius of the laser focal spot). The time dependence can be neglected here, because the laser time is an order of magnitude shorter than electron collision time scale, while still a dependence on the laser focal spot is expected.

Despite several studies and experiments were performed in the ps-ns regime, not much data is available in this fs-regime that can confirm the scaling laws predicted by theoretical models. In this paper we present the experimental results of ultrashort laser-driven EMP measurements performed on the 200 TW system VEGA 2 at the CLPU in Spain. Experimental analysis demonstrate a direct scaling between the EMP energy density and the main laser parameters such as laser Intensity *I*_*L*_ and laser energy *E*_*L*_ confirming the theoretical predictions. Numerical simulations performed by Finite Element Modelling reproduced the main EMP pulse characteristics as well as they demonstrated for the first time a theoretical framework to control the EMP-spectral emission.

The present paper is organised as follow: First, the experimental set-up is outlined; Next, the measurement techniques are presented followed by the result sections, especially highlighting the EMP scaling respect to different laser parameters; Further, numerical simulations of the target-target holder chamber system are presented; Finally, the conclusions are drawn in the final section.

**Figure 1:**
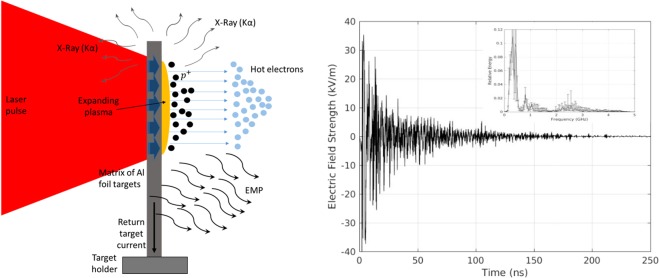
(**a**) Conceptual scheme of laser-solid target interaction with EMP generation. (**b**) Typical EMP signal detected at 30 cm from the target. Inset: normalized spectrum averaged over 23 shots.

The experimental campaign was conducted with the 200 TW (VEGA 2) laser system at CLPU Facility. The target chamber (TC) is a steel cylinder with diameter of 1.2 m and height of 0.65 m. The 40 fs long VEGA 2 pulse was focused under 20° from target normal onto a 10 *μ*m thick, 10 cm^2^ Al foil, by a F/4 gold coated parabolic mirror in a circle area with a diameter of 14 um at 1∕*e*^2^ corresponding to  ~ 7 *μ*m at the FWHM. The energy on target was estimated to be around 2.5 J giving a peak laser intensity of  ~10^20^ W/cm^2^. The Al foil was placed in a 10 × 10 array supported by 3 axes motorised holder. The conceptual scheme of the laser target interaction is shown in Fig. 1(a). Among the results of laser-target interaction, EMP’s have been measured inside the vacuum chamber by a commercial antenna and two Moebius probes placed respectively at the TCC and 30 cm far from TCC. Measurements were performed for two different polarizations, i.e. vertical and horizontal with respect to the target holder horizontal support. In Fig. 1(b) a typical EMP-signal is shown in kV/m with its spectrum in arbitrary unit in the caption. The use of calibrated cables and connectors allow us to eliminate from the measurements unwanted additional errors and uncertainty’s. For pico- and nano-timescale class lasers the EMP strength scales quasi-linearly with the delivered energy on target. However, no studies are available for fs-class lasers with multi-Joule pulse energies. So in this experimental campaign we have investigated laser energies above this limit.

**Figure 2:**
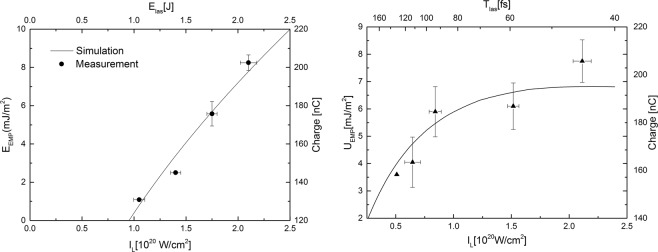
The EMP energy density as function of the laser intensity by: (left) changing the laser energy and (right) changing the laser pulse duration. The curves present the total charge deposited on target as function of the intensity obtained by computer simulations. The default input parameters of the simulation are given to be: *E*_*las*_ = 2.5*J*, *T*_*las*_ = 40 fs, Material Thickness *e*_*tar*_ = 10 *μm*, laser absorption *C*_*abs*_ = 0.4 and focal spot diameter *d*_*tart*_ = 14 *μm*.

We found the maximum measured EMP-strength amplitude, i.e. 35 kV/m, not an optimal characterisation criterion due to the ultrashort nature of the laser pulse peak, which is significantly shorter than the 50 ps sample interval of the oscilloscope. Therefore, we have chosen to characterise the EMP by its pulse energy density, which is as conservative quantity much less affected by the bandwidth of the equipment. The energy density is given by:

1 $${U}_{EMP}=(1/{T}_{0})\int {E}_{t}^{2}/{Z}_{0}dt$$

where *E*_*t*_ is the electric field given in units of *V*/*m*, *Z*_0_ the impedance of free space in units of *Ω* and *T*_0_ the EMP pulse duration given in units of second. In Fig. 2 the EMP energy density is plotted as function of the experimentally investigated laser intensities both by changing the laser energy (left) and changing the laser pulse duration (right). All error bars presented in this article were obtained by computing the standard deviation over the sample set. The black curves show the total deposited charge on target after laser interaction obtained by numerically simulations with the Fortran90 program ChoCoLaT2^28^. This program computes the time evolution of the electron cloud parameters, the evolution of the ejection current distribution and the evolution of the two contribution of the potential barrier.

The left graph demonstrate a linear scaling between *E*_*EMP*_ and *I*_*L*_ confirming the predictions of the “full ejection regime” model. From Fig. 2 one can observe a proportionality factor of 2 when we change the laser energy. This is also clearly visible from the charge simulations which show also a linear dependence as function of the pulse energy.

The graph at the right side show the EMP scaling as function of the laser pulse length. Despite of relative large error bars for the highest intensities, a clear signature of linear scaling was found up to a pulse duration of 100 fs, i.e. inversely with the laser intensity. This was confirmed by computer simulations of the accumulated charge on target. Furthermore, computer simulations show a maximum of deposited charge on target around a laser pulse duration around 50 fs after which the deposited charged is decreasing, which correspond well with the Eclipse experiments with low energy pulses^28^.

**Figure 3:**
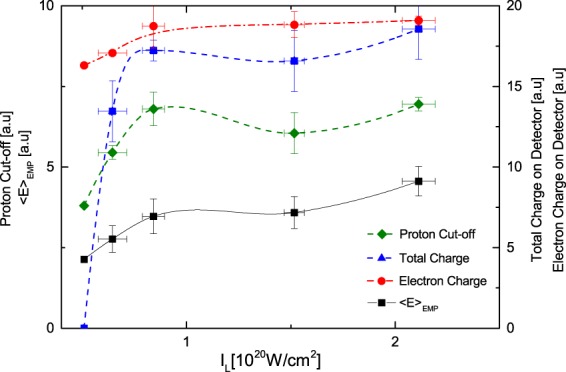
Plot of the proton cut-off energy shown by the dashed curves with green diamonds, total measured charge on detector shown by dotted curve with blue triangles, electron charge shown by dash-dot curve with red circles and the EMP Energy, shown by the black curve, with squares as function of the laser pulse intensity.

To validate the above mentioned results in Fig. 3 the EMP pulse energy is compared with complementary measurements performed during the experiment among which: (i) the emitted charge, i.e. electron and ions, shown by the dotted curve with blue triangles (measured by the pin-diode); (ii) cut-off proton energy shown by the dashed curve with green diamonds (measured by the MCP); and (iii) the electron charge, shown by dot-dash curve with red circles derived from the *K*_*α*_ emission (measured by a X-ray CCD) as a function of the laser intensity^29^. All the above quantities show a direct relation with laser intensity and with laser energy (assuming in this regime the effect of the laser duration is negligible).

### Analysis of the frequency modes and numerical simulations

**Figure 4:**
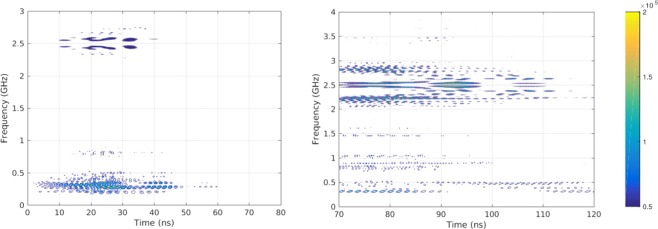
Octave wavelet analysis of the EMP from 0 to 80 ns (left side) and from 70 to 120 ns (right side). The x-axis the time is given at the y-axis the frequency. Dark blue colors correspond to high intensity frequency modes.

The time dependence of the EMP was investigated by an octave wavelet analysis (See Fig. 4). Wavelet analyses are more suitable for the analyzes of pulsed, chaotic phenomena such as earth quakes, stock market and now for the first time as tool for the analysis of EMP’s. In Fig. 4 (left) the temporal evolution is given from 0 to 40 ns. One can observe three frequency modes determined by the target-target holder-chamber system: one around 222 MHz, one around 899 MHz, and a third around 2.5 GHz. This is in well agreement with our expectations, namely that the length of the target holder *L*_*T*_ should fulfill *L*_*T*_ = *Nλ*/4, where *N* is an integer number and *λ* for the wavelength. From this thumb of rule, taken from antenna theory, we didn’t expect to find any frequency mode below 115 MHz which is indeed confirmed by the experimental obtained frequency spectrum shown in the inset of Fig. 1. If we look further in time between 70 ns and 100 ns, shown at Fig. 4 (right), one can clearly see that the low and high frequency modes are mixed leading to the creating of sub-bands. For instance the central frequency of 2.5 GHz is mixed with the frequency mode of 222 MHz.

**Figure 5:**
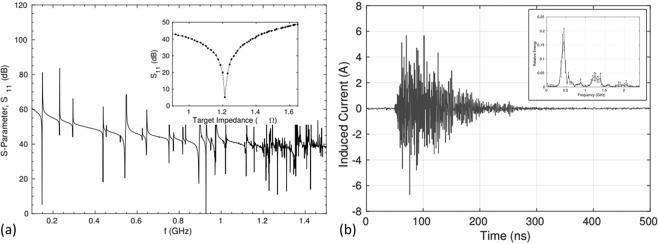
(**a**) Reflecting coefficient as function of the frequency. Inset: target impedance matching with reflection coefficient *S*_11_ as function of target impedance for f = 148 MHz. (**b**) Typical induced current into stepper motor phase line (shot 29 at 8/02/2018). Inset: relative energy of frequency modes injected into motor.

In order to obtain the corresponding eigenmodes with the measured frequency modes we performed FEM simulations with the commercial software COMSOL. In previous studies, the lowest eigenfrequency of the target chamber (TC) was obtained by modeling the chamber as an RF-cavity. These studies focused on ns-scale laser systems, limiting the frequency spectrum to the sub-GHz frequency band. In this work we investigate the utilization of fs-scale laser pulses, which greatly increases the potential bandwidth of the EMP-pulse. In this work we extended the existing models^20,22^ by including the target holder (TH) acting as a mono-pole antenna between the target chamber center (TCC) and the center of the optical breadboard. The laser target (LT) was modeled as a current source with characteristic current (*I*_*tar*_) and impedance *Z*_*tar*_^30^. The impedance of the LT with the TC was matched for the lowest eigenfrequency mode at 148 MHz, by minimizing the scatter parameter (reflection coefficient) *S*_11_ as a function of the *Z*_*tar*_ (Inset Fig. 5).

**Figure 6:**
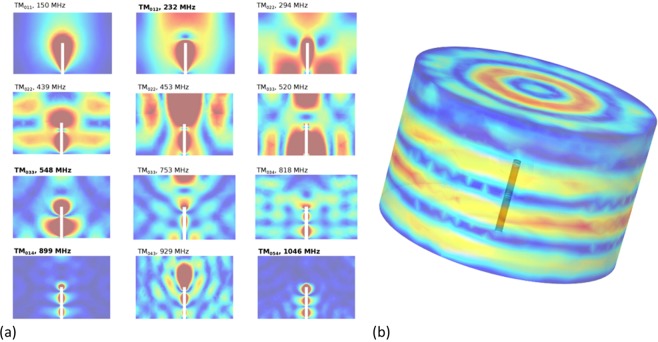
(**a**) Obtained frequency modes corresponding to the minima of the scattering parameter as shown in Fig. 5. The target holder is visible by the white rectangle at center while the target chamber is modelled as a cylinder. (**b**) One of the eigenmodes corresponding to eigenfrequency 818 MHz obtained by FEM simulation. The figure shows the electrical field strength at the surfaces. The dark rectangle in the center represents the target holder. Red colors correspond to high field strengths.

Frequencies with a low *S*-value, i.e. reflection coefficient, correspond to high RF-emission modes. The advantage of this approach is that we can limit the eigenmodes to the transversal modes of the target holder, corresponding to an Alternating Current (AC) through the target holder. The corresponding eigenmodes are given in Fig. 6 (left) with a three dimensional presentation of the electrical field on the right side. In order to obtain the strength of a given frequency mode, for a given *Z*_*tar*_, we calculated the scattering parameter ’*S*_11_’ of the cavity mono-pole system as function of the frequency as shown in Fig. 5. Around 148 MHz and 929 MHz two distinct modes are visible, corresponding to the two peaks detected in the experimental spectrum as shown in Fig. 5. Frequency modes above 1 GHz were omitted due to simulation constrains.

With respect to EMP mitigation two different strategies are predominantly being applied in the field: (1) electrical isolating the target from the target-holder, and (2) supplying a conductive path from target towards the ground. We demonstrate here that both strategies may be valid mitigation strategies in the given experimental case by plotting the scattering parameter as function of the target impedance shown in the inset of Fig. 5(b). One can observe a distinct minimum of the scattering parameter around 1.21 Ω. This means that if a target is designed (including stalk, etc.) such the impedance mismatch the target-holder vacuum chamber system, one can reduce the generated EMP significantly for certain target frequency. Note that a change of the impedance of the target of around 20% (see inset Fig. 5(a)) lead to a change in EMP emission of 40dB. This may explain why special target designs as presented in ref. ^31^ are successful in controlling and mitigating the generated EMP, hence protecting the diagnostics against the EMP.

One of the motivations of the investigation and characterization of EMP’s is the destructive impact such EMP’s have on diagnostic devices and actuators, e.g. motor controllers and cameras. During the experimental campaign we probed the current induced in the phase wires between the motor controller (PI-Micos) and stepper motor (VT80) of the target holder z-axis by a Rohde and Schwartz EZ-17 current probe. In Fig. 5(b) one can see that the induced current reaches values up to 6 A, which is several times larger than the maximum supported driving current, i.e. less than 1.2 A, of the stepper motor controller. The issues connected to this problem, i.e. the current-induced EMP’s causes the devices’ freezing their operation, a side-effect which requires a hard reset of the controller at every single shot limiting possible operation at High repetition rate. In addition it can damage the stepper motor phase driver of the controller after a few times and as such limit the operational repetition rate of ultra-short, PW-class laser solid-density experiments^8^.

### Conclusions

We have experimentally investigated the scaling of EMP in the High Intensity, Ultrafast laser matter interaction regime. We have investigated the scaling of the EMP energy as function of intensity by varying the laser pulse duration and energy. We have demonstrated that the EMP energy is scaling linear with the laser energy in the “Full ejection regime” as predicted in^4^. Furthermore, there was found that the EMP energy is increasing inversely with the laser pulse duration for the investigated parameter range. We have validated our results by complementary measurements of proton charge and cut-off energy as well as X-ray K_*α*_ radiation, which is related with accelerated electron charge. The EMP energy was found to be a good indicator for the quality of laser interaction as high EMP energy corresponded to a high proton cut-off energy and a high charge flux. At last we have performed numerical simulations to estimate the stationary eigenmodes corresponding to the measured frequencies and demonstrated that the EMP spectrum can be obtained by finite element modelling. This may enable to control the emitted frequency spectrum by tuning and engineering the impedance of target-target holder for reduced EMP generation for a given frequency bandwidth of interest in ultra-high intensity, laser-plasma experiments. This may maximise the operational repetition rate of these experiments by matching them to the repetition rate of the laser, at upcoming large-scale laser facilities such as the 2 PW, 10 Hz laser system to be installed at ELI-ALPS.

## Methods

### Experimental set-up and targetry

**Figure 7:**
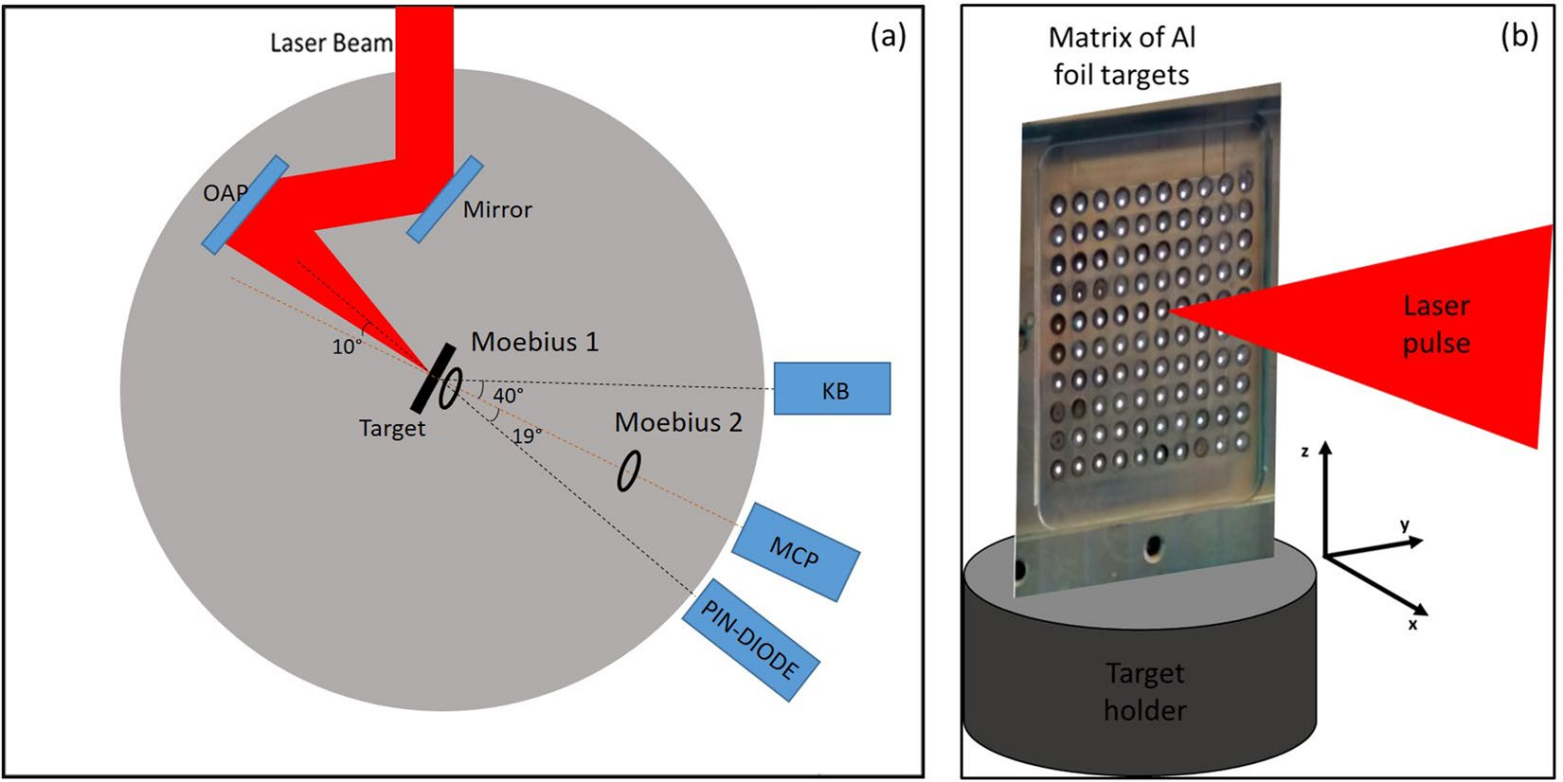
(**a**) Experimental setup: the red beam shows the laser path towards the focusing parabola and laser target. Two Moebius probes (Moebius 1 and Moebius 2) are inserted at 2.5 cm and 30 cm from the TCC respectively. The ToF detector and the Kalfa imaging system were placed outside the target chamber at 40 and 10 degree respectively with respect to the target normal. (**b**) The pinhole matrix of Al foil targets.

The experimental campaign is conducted at the 200 TW (VEGA 2) laser system at CLPU, Spain. The target chamber (TC) is a steel cylinder with diameter of 1.2 m and height of 0.65 m. The schematics of the experimental set-up is shown in Fig. 7(a). The laser beam with a diameter of 10 cm is focused by a F/4 gold coated parabolic mirror in a circle area with a diameter of 14 um at 1∕*e*^2^ corresponding to  ~7 *μ*m at the FWHM. The energy on target is up to 3.5 J with a pulse length of down to 30 fs FWHM giving a peak laser intensity of  ~10^20^ W/cm^2^ ^29^. During the experiment the laser pulse duration has been changed in a continuous way by tuning the chirp of the compressor while the laser energy has been tuned by both changing the amplification chain and applying a lambda/2 wave-plate together with a polarizer to the main laser beam before the compressor. The VEGA 2 laser beam was focused on a 10 *μ*m thick, 10 cm^2^ square Al foil placed in a 10 cm  ×  10 cm pinhole matrix (Fig. 7(b)) mounted on a motorised (x,y,z)-stage. Each pin-hole position was aligned and focused individually prior to the start of the experiment with the corresponding coordinates stored and retrieved during the experiment, for faster repositioning of the targets.

### Diagnostics

During the experiment the laser pulse duration has been changed in a continuous way by tuning the chirp of the compressor while the laser energy has been tuned by both changing the amplification chain and applying a lambda/2 wave-plate together with a polarizer to the main laser beam before the compressor. Laser plasma interaction was monitored by transporting (with a Kirk-Patrick-Baez microscope) and measuring (with a X-ray CCD camera) X-ray K_*α*_ radiation emitted from relativistic electron interaction with the target; TNSA ions charge generation has been monitored by using a pin-diode detector (PIN diode) and a Multi Channel Plate (MCP) operating in Time of Flight mode. The typical angular aperture of the ion beam was shown to be between 20 and 25 degrees. The PIN diode and the MCP were placed in the target rear direction and at a distance of 2.1 m from the target at an azimuth angle respectively of 5 and 30 degrees and a polar angle of 19 and 0 degrees with respect to the target normal axis (see the Fig. 5 of ref. ^29^). When the PIN diode collector is hit by the ions it is charged and a current signal is generated, this can be read on the oscilloscope as a time dependent voltage signal proportional to the charge. From the time dependent response signal of PIN diode, after some mathematical passages, we extracted the charge signal. The MCP operated in time domain configuration which enabled to measure the fastest ions energy. More details about particle diagnostics can be found in ref. ^29^ which results were obtained during the same experimental campaign.

The Electromagnetic Field above target was measured with a calibrated R&S Moebius probe (labeled as ‘Moebius 1’ in Fig. 7(a)) mounted 2.5 cm above target. The Field 30 cm from target in the horizontal plane of TCC (labeled as ‘Moebius 2’ in Fig. 7(a)) was measured by a custom made Moebius probe of type king type because of its flat frequency response at high frequencies. The diameter was chosen to be 2.5 cm. The signal was acquired by a R&S RTO 2044 oscilloscope with a bandwidth of 4 GHz and a sampling rate of 20 GSamples/s. The scope was triggered by the facility wide triggering system. The probes were calibrated at the RF-lab of the university of Gyor, Hungary.

In both case the raw data were corrected against cable and frequency dependent connector attenuation.

### Numerical simulations

We performed Finite Element simulations with the commercial software COMSOL 5.3. The target chamber was modelled as a cylinder with a diameter of 120 cm and height of 65 cm. The target holder was modelled as a cylinder between the TCC and the middle of the bottom of the cylinder. The impedance of the target itself was modelled as lumped port allow us to inject a RF-current into the target holder.
